# Supplementary material for: WordCommentsAnalyzer: A windows software tool for qualitative research
Source: F1000Res. 2018 Sep 4;7:536. Originally published 2018 May 3. [Version 2] doi: 10.12688/f1000research.14819.2 (PMC6137411; doi:10.12688/f1000research.14819.2)
Supplement: Supplementary file 3 [file f1000research-7-17614-s0002.tgz › b2a79496-4ada-47c4-9682-a99ce867836d.zip!/Eight Iranian Health Journals/Journal of Health System Research _19.docx]

© Journal of Health System Research . This work is licensed under CC BY-NC 4.0 (https://creativecommons.org/licenses/by-nc/4.0/).

Volume & Issue: ویِژه نامه تغذیه؛ 1392

Title: پپتید-1 شبه گلوکاگون (1-GLP) در تنطیم قند خون و درمان دیابت نوع دو: مرور سیستماتیک

http://hsr.mui.ac.ir/index.php/jhsr/article/view/1366

چکیده پپتید شبیه به گلوکاگون-1 (GLP-1) از جمله اینکرتین هورمونهاست که پس از هضم غذا، از روده ترشح شده و سبب افزایش ترشح انسولین و تنظیم قند خون میشود. همچنین قادر به مهار مرگ سلولی و افزایش تکثیر سلولهای بتای پانکراس میباشد.هدف از مطالعه حاضر، مروري بر شواهد موجود در زمينه اثرات GLP-1 در ترشح انسولین ، تنظیم قند خون و کاربرد آن در درمان دیابت نوع 2 میباشد.در این بررسی، مطالعات مورد- شاهدي، كوهورت و مشاهده اي و مروری درنظر گرفته شد و بررسی با استفاده از موتور جستجوي PubMed، بين سال هاي 1987 تا 2012 و نيز استفاده از كليد واژه هاي  glucagon-like pepetide-1, GLP-1،, "type 2 diabetes", "type II diabetes" NIDDMانجام گردید و در كل 25 مقاله مورد استفاده قرار گرفت. GLP1 به دو طریق مستقیم و غیرمستقیم بر افزایش میزان انسولین و کاهش قند خون اثر دارد. از یک سو از طریق گیرنده های  موجود بر سلولهای پانکراس بطور مستقیم سبب افزایش بیان ژن انسولین و سنتز آن شده واز سوی دیگر، با اثر  بر گیرنده¬های خود بر روی سلولهای β پانکراس و به طور غیرمستقیم از طریق سیستم عصب واگ و ورید پورتال کبدی باعث افزایش ترشح انسولین خواهد شد. تحقیقات  نشان می¬دهد که در دیابت نوع II، ترشح GLP-1 دچار اختلال می¬شود، بنابراین افزایش و بهبود سطح GLP-1 خون می¬تواند سبب افزایش انسولین و تنظیم گلوکز خون شود. امروزه دو نوع از داروهای افزایش دهنده اثر GLP1 بصورت خوراکی و تزریق زیرجلدی مورد استفاده قرار میگیرد.

Title: عوامل پخت تاثیرگذار بر محتوای فلزات سنگین در برنج

http://hsr.mui.ac.ir/index.php/jhsr/article/view/603

مقدمه: در سال‌های اخیر، آلودگی برنج در برخی از نقاط جهان موجب کاهش ایمنی مصرف آن و بروز بحرانی در تولید و تجارت آن شده است. در ایران نیز گزارشات مختلفی از آلودگی تولیدات داخلی به فلزات سنگین ارایه شده است. اما غلظت فلزات سنگین طی فرآوری و پخت برنج تغییر می‌کند. هدف از این مقاله مروری، بررسی عوامل موثر در تغییر غلظت فلزات سنگین در پخت برنج می‌باشد. روش‌‌ها: بدین منظور، با جستجو در منابع علمی معتبر 51 مقاله از سال‌های 1994 تا 2012 فراهم شد و با بررسی خلاصه مقالات، 18مقاله کاملا مرتبط با موضوع استخراج گردید. یافته‌ها: : مجموعه عوامل روش پخت، غلظت فلزات در آب پخت، دمای آب، نوع عنصر سنگین، ظرف پخت، واریته برنج و حجم برنج خام عوامل اصلی مورد بحث در تغییر محتوای عناصر سنگین طی پخت برنج می‌باشند که در این مطالعه تاثیر آنها به تنهایی یا همراه با سایر عوامل مورد بحث قرار گرفت. روش پخت اگرچه یکی از مهمترین عوامل است اما اثر آن بسته به نوع عنصر متفاوت است. غلظت فلزات در آب مورد استفاده در پخت عامل مهمی در افزایش این عناصر در غذاهایی است که به فرم فرنی تهیه می‌شوند. اثر نوع ظرف در میزان غلظت فلزات سنگین باقی مانده در برنج جزئی است. با این حال،پخت در ظرف آلومینیوم باعث افزایش این عنصر در برنج شده است. تفاوت در واریته برنج، حجم برنج خام مورد استفاده و میزان غلظت اولیه فلز سنگین در برنج خام نیز درشرایط یکسان پخت، اثر جزئی در میزان باقیمانده عناصر سنگین در برنج پخته شده دارند. نتیجه‌گیری: با توجه به اثر نتایج برخی از مطالعات اخیر از حدود غلظت بالای برخی عناصر سنگین در انواع برنج موجود در بازار ایران و نتایج مطالعه حاضر از اثر عوامل پخت بر محتوای فلزات سنگین در برنج و با در نظر گرفتن روش‌های خاص تهیه برنج در کشور و سهم قابل توجه برنج در رژیم غذایی جامعه، می‌توان گفت که در این زمینه خلاء اطلاعاتی قابل توجهی وجود دارد. انجام مطالعات در این رابطه در ارزیابی خطر دریافت فلزات سنگین از طریق مصرف برنج مستقیما مورد استفاده خواهد بود.

Title: بررسی اثر روش‌های رایج پخت در جامعه ایرانی بر برخی از شاخص‌های تغذیه‌ای گوشت شتر

http://hsr.mui.ac.ir/index.php/jhsr/article/view/1177

مقدمه: گوشت شتر نسبت به سایر منابع گوشت قرمز، حاوی ماده چرب کمتری است در حالی که از حیث سایر مواد مغذی با گوشت سایر دام‌ها برابری می‌کند. این مطالعه با هدف بررسی اثر سه روش پخت رایج در جامعه ایرانی بر تغییرات میزان ماده چرب و تغییرات میزان آهن در این گوشت صورت گرفت. روش‌ها: سه روش آب پز کردن، سرخ کردن و کباب کردن هر کدام در دما و زمان مشخص و مطابق با عادت جامعه ایرانی فراوری شد. رطوبت، خاکستر و افت وزن پخت با استفاده از روش‌های استاندارد تعیین گردید. چربی از روش فلش و میزان آهن در نمونه‌های گوشت فراوری شده با روش جذب اتمی تعیین شد. آهن هم با روش اسپکتروفتومتریک و قرائت جذب در طول موج 630 نانومتر مشخص گردید. یافته‌ها: نمونه‌های آب پز شده، کبابی و سرخ شده به ترتیب بیشترین افزایش ماده چرب را نشان دادند. تغییرات خاکستر نشان‌دهنده موثر بودن روش کباب کردن و سرخ کردن در حفظ املاح و مواد معدنی بوده در حالی که بیش از 40% از املاح در ماده خشک طی روش آب پز کردن از دست رفت. تغییرات آهن کل و آهن هم نشان داد که علی‌رغم عدم وجود اختلاف معنی‌دار (05/0 <P) میان میزان آهن کل در ماده خشک نمونه‌های فراوری شده به سه روش، در کباب کردن بیشتر میزان آهن هم حاصل شده است. نمونه آب پز و نمونه سرخ شده از این نظر با اختلاف قابل توجهی درسطوح بعدی قرار گرفتند. نتیجه‌گیری : با اعمال روش کبابی کردن ضمن عدم افزایش قابل توجه چربی در ماده خشک بیشترین میزان آهن قابل جذب در گوشت پخته شده حفظ می‌شود. مدت زمان پخت کمتر و دمای پایین‌تر در مرکز قطعات گوشت (80 درجه سانتیگراد) احتمالا در نتایج مشاهده شده موثر بوده است.

Title: بررسی ارتباط الگوی غذایی شبه غربی و خطر ابتلا به سندرم متابولیک در بزرگسالان شهر خرم‎آباد

http://hsr.mui.ac.ir/index.php/jhsr/article/view/1110

مقدمه: هر چند ارتباط غذاها و مواد مغذي با سندرم متابوليك تا حدودي شناخته شده است، مطالعه الگوهاي غذايي و ارتباط آن با سندرم متابولیک فواید بیشتری نسبت به بررسی ارتباط منحصراً یک یا چند ریز مغذی دارد؛ بنابراين مطالعه حاضر با هدف تعیین ارتباط الگوهاي غذايي شبه غربی با سندرم متابوليك در شهر خرم‌آباد انجام شد. روش‌ها: در اين مطالعه مقطعي، 973 نفر (237 مرد و 736 زن) از ساکنین شهر خرم‌آباد به روش نمونه‌گيري خوشه‌اي چند مرحله‌اي و به صورت تصادفي سیستماتیک انتخاب شدند. دريافت‌هاي غذايي افراد ارزيابي، وزن و قد اندازه‌گيري و نمايه توده بدن محاسبه شد. نمونه خون سياهرگي جهت اندازه‌گيري سطح متغيرهاي متابوليكي جمع‌آوري و فشار خون اندازه‌گيري شد. سندرم متابوليك طبق معیارهای AHA (انجمن قلب آمریکا) تعريف شد. یافته‌ها: در این مطالعه نشان داده شد افرادی که در بالاترین پنجک الگوی غذایی شبه غربی قرار داشتند، در مقایسه با افراد پایین‌ترین پنجک، شانس بیشتری برای ابتلا به سندرم متابولیک(نسبت شانس 21/2 و فاصله اطمینان 95%: 42/1 تا 44/3) داشتند. نتیجه‌گیری: يافته‌هاي مطالعه حاضر نشان مي‌دهد که الگوی غذایی شبه غربی (گوشت احشا، مارگارین، قهوه، خشکبار، شیرینی و دسر، چاشنی‌ها، نوشابه، متفرقه، گوشت قرمز و گوشت فرآوری شده) خطر سندرم متابولیک را افزایش می‌دهد.

Title: اثر مصرف مکمل اسکوربیک اسید برتوان هوازی و بی هوازی بازیکنان هند بال

http://hsr.mui.ac.ir/index.php/jhsr/article/view/1056

مقدمه:اسکوربیک‌اسید یا ویتامین C یکی از شناخته شده‌ترین ویتامین‌های محلول در آب است.چون تامین انرژی اضافی لازم برای ورزش از طریق غذای مصرفی با افزایش متناسب دریافت ویتامین  Cهمراه نیست. ورزش شدید می‌تواند باعث استرس اکسیداتیو درسلول‌ها، درد عضلانی و افت عملکرد ورزشی شود. کارآیی ویتامین C به عنوان عامل آنتی‌اکسیدانی و مؤثر بودن آنها در عملکرد ورزشی در بررسی‌های انسانی اندکی مورد مطالعه قرار گرفته است. بنابراین ما اثرات مصرف زیاد اسکوربیک‌اسید را برتوان هوازی و بی‌هوازی بازیکنان هندبال مورد بررسی قرار دادیم.  روش‌ها : 14 زن ورزشکار تعلیم دیده بازیکن هندبال، در دامنه سنی 33-19 سال، در این بررسی ثبت نام و به طور تصادفی در دو گروه (دریافت‌کننده 500 میلی‌گرم ویتامین  Cاضافی یا دارونما)، قرار گرفتند و توان هوازی و بی‌هوازی در آغاز و پایان بررسی ارزیابی و بین گروه‌ها و داخل گروه‌ها با استفاده از آزمون t انجام شد. یافته‌ها : 14 نفر از شرکت‌کنندگان یک هفته مداخله درمانی را کامل کردند. هیچ تغییر قابل‌توجهی در عملکرد ورزشی هوازی و بی‌هوازی در بین گروه‌ها در مقایسه با گروه دارونما وجود نداشت. اما تغییر معنی‌دار درون گروهی درعملکرد ورزشی هوازی در گروه مصرف‌کننده ویتامین) C 05/0 (P<وجود داشت. نتیجه‌گیری: مصرف روزانه 500 میلی‌گرم ویتامین Cبه مدت یک هفته در ورزشکاران زن، هیچ تغییر قابل‌توجهی در سطح عملکرد ورزشی هوازی و بی‌هوازی در مقایسه با گروه دارونما ایجاد نکرد.

Title: ارتباط چربی رژیم غذایی با پروفایل لیپیدی و سطح CRP سرم در زنان مبتلا به بیماری مالتیپل اسکلروز (MS)

http://hsr.mui.ac.ir/index.php/jhsr/article/view/978

مقدمه: بیماری  Multiple Sclerosis(MS) از شایع‌ترین بیماری‌های سیستم عصبی در انسان به شمار می‌آید. MS یک بیماری مزمن پیشرونده و دمیلینه‌کننده سیستم عصبی مرکزی با سیر متفاوت و پیش‌آگهی نامشخص می‌باشد. تغذیه و رژیم غذایی از جمله عوامل مؤثر در پیشگیری و درمان این بیماری می‌باشد. اما نتایج مطالعات در این زمینه متناقض می‌باشد. مطالعه حاضر با هدف بررسی وضعیت الگوی چربی مصرفی در بیماران مبتلا به MS و ارتباط آن با سطح فاکتور التهابی CRP انجام شد. روش‌ها: مطالعه حاضر به روش مقطعی بر روی 35 زن مبتلا به MS مراجعه‌کننده به کلینیک MS بیمارستان کاشانی شهر اصفهان دارای معیارهای ورود انجام شد. ویژگی‌های دموگرافیک افراد از طریق پرسشنامه جمع‌آوری شد. فاکتورهای بیوشیمیایی خون بیماران (کلسترول توتال، تری‌گلیسرید، HDL، LDL و CRP) با روش‌های استاندارد موجود اندازه‌گیری شد. رژیم غذایی افراد با استفاده از پرسشنامه نیمه کمی بسامد خوراک با تمرکز بر موارد مربوط به چربی‌های رژیم غذایی ارزیابی شد. یافته‌ها: میانگین سنی افراد شرکت‌کننده در مطالعه 14/8±04/33 بود. همبستگی بین نوع چربی دریافتی (SFA، PUFA، MUFA) و لیپیدهای خون و CRP پس از کنترل عوامل مداخله‌گر از قبیل سن، وزن و کل انرژی دریافتی در بین هیچ کدام از متغیرها از نظر آماری معنی‌دار نبود. پس از انجام رگرسیون خطی چند متغیره در هیچ کدام از مدل‌ها ارتباط معناداری از نظر آماری مشاهده نشد. نتیجه‌گیری: در این مطالعه ارتباط معنی‌داری بین نوع چربی غذایی دریافتی با افزایش ریسک فاکتورهای بیماری MS مشاهده نشد. ارتباط بین تغذیه و بیماری MS نیاز به تحقیقات بیشتر دارد.

Title: بررسی ارتباط نوشیدن چای با تراکم املاح استخوانی در زنان یائسه مراجعه‌کننده به مرکز پوکی استخوان شهر اصفهان

http://hsr.mui.ac.ir/index.php/jhsr/article/view/979

مقدمه: مصرف چای در جامعه ما عمومیت داشته و به مراتب بیشتر از قهوه و نوشیدنی‌های دیگر استفاده می‌شود. بررسی‌های متعددی ارتباط قهوه با تراکم املاح استخوانی (BMD) را ارزیابی کرده‌اند اما ارتباط بین مصرف چای و تراکم استخوان کمتر ارزیابی شده است. با توجه به شیوع بالای پوکی استخوان در کشور و تغییرات الگوی جمعیتی که با افزایش افراد میانسال و مسن همراه است، بررسی ارتباط بین نوشیدن چای و تراکم املاح استخوانی می‌تواند ارزشمند باشد. روش‌ها: این مطالعه به صورت مقطعی توصیفی - تحلیلی انجام شد. تعداد 87 نفر از جمعیت زنان یائسه مراجعه‌کننده به مرکز پوکی استخوان اصفهان به صورت نمونه‌گیری تصادفی ساده وارد مطالعه شدند. از طریق پرسشنامه اطلاعات فردی از افراد گرفته شد. برای ارزیابی میزان دریافت کلسیم و ویتامین D پرسشنامه بسامد غذایی به این منظور تهیه شد. تراکم املاح استخوانی با روش DEXA در دو ناحیه لگن و ستون فقرات اندازه‌گیری شد. یافته‌ها: میانگین و انحراف معیار سن و شاخص توده بدنی (BMI) زنان شرکت‌کننده در مطالعه به ترتیب 48/7 ± 1/58 سال و 38/4 ± 4/28 بود. مدل رگرسیون خطی چندگانه نهایی نشان داد که میزان مصرف چای مستقل از عوامل سن، BMI، فعالیت بدنی، مصرف قهوه، دریافت روزانه کلسیم از رژیم غذایی، دریافت مکمل کلسیم و دریافت روزانه ویتامین D از رژیم غذایی، با افزایش تراکم املاح استخوانی در ناحیه لگن (22/53β=  , 001/0 >p) و ناحیه ستون فقرات (46/55β= , 001/0 >p) در ارتباط بود. نتیجه‌گیری: بر اساس یافته‌های مطالعه ، مصرف چای با افزایش BMD در ارتباط بود که این ارتباط مستقل از عوامل سن، BMI، فعالیت بدنی، و سایر مخدوش کننده‌های مؤثرمی‌باشد.

Title: بررسی اثر امگا3 در مقایسه با پلاسبو بر تغییرات وزن و دور کمر، دور باسن و ارتباط آن با ویسفاتین در بیماران دیابتی نوع2

http://hsr.mui.ac.ir/index.php/jhsr/article/view/912

مقدمه: چاقی و بالا بودن اندازه دور کمر به باسن ( نوع آندروئید) در شیوع بیماری‌های قلب و عروق و دیابت نوع 2 نقش دارد. افزایش بافت چربی به خصوص چربی احشایی وابستگی کاملی به عوامل خطرآفرین بیماری‌های قلبی- عروقی چون عدم تحمل گلوکز، هیپرلپیدمی، پرفشاری خون و همچنین به عنوان عامل زمینه‌ساز در دیابت و سندرم متابولیک نقش دارد. در بیشتر موارد مقاومت به انسولین و دیابت نوع دو با چاقی همراه است . روش‌ها: تعداد 71زن مبتلا به دیابت نوع 2به دو گروه تحت درمان با کپسول امگا3 وگروه کنترل با کپسول‌ دارونما قرار گرفتند. سن، قد، وزن، دور کمر از طریق مشاهده و پرسشنامه گرفته شد. بیماران پس از 8 هفته از نظر میانگین موارد فوق ارزیابی شدند. اطلاعات به دست آمده توسط نرم افزار SPSS  مورد تحلیل قرار گرفت . یافته‌ها میانگین وزن بیماران،BMI، دور کمر، دور باسن و نسبت آنها در 2 گروه قبل و بعد از مداخله تفاوت معنی‌داری نداشت (P>0.05) اما میانگین تغییرات وزن بیماران، BMI، دور کمر، دور باسن و نسبت آنها در 2 گروه تفاوت معنی‌داری داشت (P<0.001) . نتیجه‌گیری: از یافته‌های به دست آمده می‌توان نتیجه گرفت که امگا3 باعث کاهش وزن بیماران گردیده است همچنین BMI و WHR کم شده است ولی با توجه به اینکه مدت انجام مطالعه کوتاه بوده است این تغییرات در بعد از مداخله معنی‌دار نبود اما میانگین تغییرات آنها تفاوت معنی‌داری داشته است باید تحقیقات جدیدی با مدت بیشتر انجام گردد.

Title: میزان فراوانی کلستریدیوم دیفیسیل در فرایند تولید فراورده گوشتی همبرگر

http://hsr.mui.ac.ir/index.php/jhsr/article/view/1264

مقدمه: کلستریدیوم دیفیسیل به عنوان عامل اسهال ناشی از مصرف آنتی بیوتیک وکولیت با غشای کاذب است. محصولات گوشتی به عنوان یکی از منابع احتمالی انتقال کلستریدیوم دیفیسیل به انسان محسوب می‌شوند. این مطالعه با هدف بررسی فراوانی  کلستریدیوم دیفیسیل در فرایند تولید همبرگر با استفاده از روش کشت و آزمون بیوشیمیایی انجام گرفت.  روش‌ها: در این مطالعه توصیفی- تحلیلی، 211 نمونه شامل مواد اولیه متشکله و محصول نهایی همبرگر سه کارخانه فراورده گوشتی دارای گواهینامه HACPP و چهار کارخانه فاقد آن در استان اصفهان در فاصله زمانی تیر تا دی ماه1391 جمع‌آوری گردید. نمونه‌برداری با سواب از سطوح ماشین‌آلات فرایند تولید نیز انجام گرفت. نمونه‌ها در محیط مایع کلستریدیوم دیفیسیل موکسالاکتام نوروفلوکسازین"CDMN"  غنی‌سازی و سپس در محیط CDMN آگار به صورت بی‌هوازی کشت داده شدند. پرگنه‌های مشکوک کلستریدیوم دیفیسیل در محیط بلاد آگار درشرایط بی‌هوازی دوباره کشت داده شد و با استفاده از آزمون‌های بیوشیمیایی شناسایی گردید. نتایج حاصل با نرم‌افزار SPSS و آزمون آماری  χ 2 مورد آنالیز و 05/0P   معنی‌دار در نظر گرفته شد. يافته‌ها: فراوانی کلستریدیوم دیفیسیل در کارخانجات دارای گواهینامه HACCP ،5/5% و در کارخانجات فاقد گواهینامه،14% بود. بر پایه نتایج، اختلاف معنی‌داری در شیوع باکتری طی فصول مختلف (34/0 (P= مشاهده نشد ولی شیوع کلستریدیوم دیفیسیل در کارخانجات دارای گواهینامه HACCP در مقایسه با کارخانجات فاقد گواهینامه اختلاف معنی‌داری را نشان داد (03/0 = P). نتيجه‌گيري:  نتایج این مطالعه نشان داد که احتمالا" مصرف همبرگر در انتقال کلستریدیوم دیفیسیل به انسان نقش دارد و اجرای اصول HACCP در کارخانجات تولید‌کننده فراورده گوشتی همبرگر می‌تواند در کاهش شیوع کلستریدیوم دیفیسیل موثر باشد.

Title: ارتباط مصرف حبوبات با سندرم متابولیک در زنان

http://hsr.mui.ac.ir/index.php/jhsr/article/view/1222

مقدمه: مطالعه حاضر با هدف بررسي ارتباط مصرف حبوبات و سندرم متابوليک در زنان پرستار اصفهاني انجام شد. روش‌ها: اين مطالعه مقطعي بر روي 420 پرستار زن 60-20 ساله شاغل در بيمارستان‌هاي اصفهان که به روش نمونه‌گيري خوشه‌اي چند مرحله‌اي به صورت تصادفي انتخاب شده بودند انجام شد. دريافت‌هاي غذايي معمول فرد در طي سال گذشته با استفاده از يك پرسشنامه نيمه كمي بسامد خوراك مشتمل بر ليستي از 106 غذا و قلم غذايي به همراه يك اندازه استاندارد از هر ماده غذايي ارزيابي شد. دریافت حبوبات از طریق جمع کردن مصرف عدس، ماش، نخود و انواع لوبیاها محاسبه شد. شاخص‌هاي تن‌سنجي و فشارخون طبق دستورالعمل‌هاي استاندارد ارزيابي شد. تمام شاخص‌هاي بيوشيميايي بر روي نمونه خون ناشتا اندازه گيري شدند. سندرم متابوليک بر طبق معيارهاي ATP III تعريف شد. يافته‌ها: با افزایش مصرف حبوبات تفاوت آماری معنی‌داری از نظر ابتلا به سندرم متابولیک و برخی اجزای آن مشاهده نشد، ولی افراد در بالاترین سهک مصرف حبوبات نسبت به افراد در پایین‌ترین سهک، از شیوع هایپرتری‌گلیسیریدمی (3 در مقابل 4/11%،03/0=P) و HDL پایین (1/41 در مقابل 8/51%، 09/0=P) کمتری برخوردار بودند. قبل از تعدیل اثر عامل مخدوشگر افرادی که در بالاترین سهک مصرف حبوبات قرار داشتند نسبت به افرادی که در پایین‌ترین سهک مصرف حبوبات بودند 28% ( (27/1-29/0)72/0 ) شانس کمتری برای ابتلا به سندرم متابولیک داشتند اما این ارتباط از نظر آماری معنی‌دار نبود. پس از تعدیل اثر سن و انرژی دریافتی و همین طور سایر مخدوشگرها، این ارتباط تا حدودی تقویت شد اما همچنان از نظر آماری معنی‌دار نبود. با در نظر گرفتن دریافت‌های غذایی، مشاهده شد که افراد در بالاترین سهک مصرف حبوبات نسبت به افراد پایین‌ترین سهک، از 41% ( (98/0-11/0)59/0 ) شانس کمتری برای ابتلا به سندرم متابولیک برخوردار بودند که این ارتباط از نظر آماری معنی‌دار بود، البته تعدیل بیشتر برای نمایه توده بدنی این ارتباط معنی‌دار را از بین برد ( (07/1-17/0)66/0 ). نتيجه‌گيري: يافته‌هاي اين مطالعه حاکي از وجود ارتباط معکوس معني‌دار بين مصرف حبوبات و شیوع دو جزء از اجزای سندرم متابوليک (هیپرتری‌گلیسریدمی و HDL کلسترول پایین) بود. ارتباط معنی‌داری بین مصرف حبوبات و شیوع سندرم متابولیک و دیگر اجزای سندرم متابوليک یافت نشد. انجام مطالعات آينده‌نگر براي تأييد اين يافته‌ها ضرورت دارد.

Title: ارتباط بين مصرف لبنيات با بيماري‌هاي قسمت فوقاني دستگاه گوارش

http://hsr.mui.ac.ir/index.php/jhsr/article/view/1324

مقدمه: رژيم غذايي مي‌تواند هم در بروز و هم در کاهش علایم بیماری‌های فوقانی دستگاه گوارش به خصوص سوء هاضمه و ریفلاکس مری-معدی مؤثر باشد. لبنيات از جمله گروه‌هاي غذايي که در ارتباط با بيماري‌هاي گوارش است. روش‌ها: اين پروژه در قالب طرح سپاهان بر روی کارمندان دانشگاه علوم پزشکی اصفهان به روش مقطعی انجام شد. اطلاعات در طي دو مرحله جمع‌آوري شد. در مرحله اول داده‌هاي تغذيه با استفاده از پرسشنامه بسامد غذايي و در مرحله دوم اطلاعات مربوط به اختلالات عملکردي دستگاه گوارش با پرسشنامه استاندارد ROME III جمع‌آوری شد. تعداد افرادي که اطلاعات هر دو مرحله‌ي آنها کامل بود 4763 نفر بودند. یافته‌ها: افرادی که در بالاترین رده مصرف لبنیات قرار داشتند نسبت به کسانی که در پایین رده دریافت لبنیات بودند، پس از تعدیل برای مخدوشگرهای بالقوه، 26% شانس کمتری برای ابتلا به ریفلاکس مری-معدی دارا بودند. ارتباط معکوسی بین مصرف لبنیات و سوء هاضمه عملکردی و اجزای متشکله آن نیز مشاهده شد. نتیجه‌گیری: ارتباط معکوسی بین دریافت لبنیات و بیماری‌های فوقانی دستگاه گوارش وجود دارد و در افرادی که در بالاترین رده مصرف لبنیات قرار داشتند نسبت به کمترین رده مصرف لبنیات، شیوع ریفلاکس مری- معده، سوء هاضمه عملکردی و اجزا متشکله آن کمتر بود.

Title: بررسی اثر مصرف گروه های غذایی و گلیومای بزرگسالان: مطالعه مورد-شاهدی گلیومای بزرگسالان

http://hsr.mui.ac.ir/index.php/jhsr/article/view/1577

مقدمه: سرطان به عنوان سومين علت مرگ (عامل 14٪ از كل مرگ و ميرها)، به يك معضل اساسی بهداشتي مبدل شده است. سرطان ها همانند اغلب بيماريهاي مزمن غير واگير و يا بيماري هاي واگير، قابل پيشگيري هستند. در مطالعه حاضر ارتباط میان گروه هاي غذایی با گلیومای بزرگسالان مورد بررسي قرار گرفت. روش‌ها: در این مطالعه مورد – شاهدی مبتنی بر بیمارستان، اطلاعات اجتماعی-اقتصادی، دموگرافیک، عوامل سبک زندگی، بهداشتی و دریافت‌های غذایی 128 بیمار مبتلا به انواع گلیوما تازه تشخیص مورد تایید پاتولوژیست و 256 شاهد از طریق مصاحبه و تکمیل پرسشنامه بسامد خوراک نیمه کمی (FFQ) معتبر و قابل اطمینان جمع آوری گردید. تمام آزمون‌های آماری با استفاده از نرم‌افزار SPSS نسخه 19 انجام شد. یافته‌ها: پس از تعدیل برای مخدوشگرهای بالقوه، رابطه معکوسی میان گلیومای بزرگسالان و دریافت بیشتر چای و قهوه (040/0 =P ؛72/0 - 16/0 :CI %95 ؛34/0 =OR: بالاترین سهک در مقابل پایین ترین سهک)، لبنیات (002/0 =P ؛82/0 - 18/0 :CI %95 ؛39/0 =OR : بالاترین سهک در مقابل پایین ترین سهک)و حبوبات (001/0 =P ؛86/0 - 20/0 :CI %95 ؛41/0 =OR : بالاترین سهک در مقابل پایین‌ترین سهک) مشاهده نمودیم. ارتباط معنی‌داری میان دریافت گوشت‌های فرآوری و نمک سود شده، تخم‌مرغ، میوه‌ها، سبزی‌ها، گوشت‌ها، شیرینی‌ها، روغن‌های هیدروژنه، نوشیدنی‌های صنعتی، چربی‌ها و مغزها و شانس ابتلا به گليوما مشاهده نشد. نتیجه‌گیری: نتایج ما از تاثیر احتمالی برخی گروه‌های غذایی مانند چای و قهوه، لبنیات و حبوبات در پيشگيري از گليوما در افراد بزرگسال حمایت می‌کند.

Title: اثر مکمل یاری با ویتامین D بر روی پروتئین واکنشگر با حساسیت بالاhs-CRP، پروفایل متابولیکی و استرس اکسیداتیو در زنان باردار

http://hsr.mui.ac.ir/index.php/jhsr/article/view/1356

مقدمه: افزایش فاکتورهای التهابی با پیدایش چندین عارضه در ارتباط است. با توجه به دانش ما هیچ گزارشی که نشان دهنده اثر مکمل ویتامین D بر روی hs-CRP سرم، پروفایل متابولیکی و بیومارکرهای استرس اکسیداتیو در بین زنان باردار موجود نیست. این مطالعه برای تعیین اثر مکمل یاری ویتامین D بر روی hs-CRP سرم پروفایل متابولیکی و بیومارکرهای استرس اکسیداتیو در میان زنان باردار انجام شده است. روش‌‌ها: این کارآزمایی بالینی در میان ۴۸ زن باردار، بارداری اول، با سن ۴۰-۱۸ سال که تک قلو حامله بودند و در هفته ۲۵ بارداری بودند اجرا شد. شرکت‌کنند‌گان هرکدام به صورت تصادفی برای ۹ هفته، مکمل ویتامین D (24=n) یا دارونما (24=n) دریافت کردند. نمونه خون ناشتا در ابتدای مطالعه و بعد از ۹ هفته برای اندازه‌گیری hs-CRP پروفایل متابولیکی و بیومارکرهای استرس اکسیداتیو شامل ظرفیت تام آنتی اکسیدانی پلاسما(TAC)  و گلوتاتیون تام (GSH) گرفته شد. یافته‌ها: مکمل یاری منجر به افزایش سطح ویتامین D سرم (003/0 Pgroup=) و کلسیم (01/0  Pgroup=) شد. افراد گروه مداخله، کاهش زیادی در سطح سرمhs-CRP  (01/0Pgroup=)  و افزایش قابل توجهی‌ در غلظت (TAC) پلاسمای‌ آنها در مقایسه با گروه دارونما داشتند (002/0 Pgroup=) .اثر قابل توجه حاشیه‌ای مکمل یاری با کاهش سطح تری‌گلیسیرید (06/0Pgroup=) سرم و فشار خون دیاستولیک )07/0Pgroup=) مشاهده شد. ما موفق نشدیم که اثر قابل توجهی‌ از مکمل ویتامین D بر روی گلوکز خون ناشتا، سطح انسولین سرم، فشار خون سیستولیک، پروفایل‌های لیپیدی دیگر و گلوتاتیون تام پلاسما پیداکنیم. نتیجه‌گیری: در نتیجه مکمل یاری ۹ هفته‌ای در زنان باردار منجر به کاهش سطح hs-CRP و افزایش (TAC) پلاسما، ویتامین D سرم و غلظت کلسیم می‌شود. علاوه بر این مکمل یاری منجر به اثر حاشیه‌ای بر روی فشار خون دیاستولیک (DBP) و تری گلیسیرید سرم می‌شود اگرچه نمی‌تواند بر روی پروفایل متابولیکی سرم اثری داشته باشد.

Title: تأثیر مصرف چاي‌ترش بر پروفايل ‌ليپيدي افراد مبتلا به ديس‌ليپيدمي

http://hsr.mui.ac.ir/index.php/jhsr/article/view/1026

مقدمه: ديس‌ليپيدمي مهم‌ترين علت بيماري‌های قلبي‌ عروقی در جهان است و درمان گياهي نقش مهمي در درمان ديس‌ليپيدمي دارد. با توجه به اينكه مطالعات محدودي در خصوص اثر چاي‌ترش (Hibiscus sabdariffa L.) در مدل‌هاي انساني انجام شده، این مطالعه با هدف تعیین تأثير مصرف چای‌ترش بر پروفايل ليپيدي افراد مبتلا به دیس‌ليپيدمي انجام گرفت. روش‌ها: یک مطالعه کار آزمایی بالینی تصادفي کنترل‌دار بر روی 43 نفر از بیماران مبتلا به دیس‌لیپیدمی پلي‌ژنيك بدون سابقه بیماری قلبی عروقی، دیابت، کلیوی، کبدی، تیروئیدی كه هيچگونه داروي پایين‌آورنده چربي خون مصرف نمي‌كردند، انجام گرفت. بیماران به طور تصادفی به دو گروه شاهد و تجربی تقسیم ‌شدند. به هر دو گروه در ابتداي طرح، آموزش‌هاي اصلاح سبك زندگي داده ‌شد. گروه تجربی روزانه دو ليوان چاي ‌ترش را صبح و عصر بين وعده‌هاي غذايي به مدت 90 روز مصرف نمودند که جایگزین دو لیوان از چای‌سیاه مصرفی روزانه شد. در ابتدا و روزهای 45 و 90 نمونه خون افراد بعد از 14-12 ساعت ناشتايي گرفته و پروفایل لیپیدی اندازه‌گيري شد. یافته‌ها: در گروه تجربی، تغییرات میانگین کلسترول‌تام پایه، روزهای 45 و 90 اختلاف معنی‌داری داشته و کاهش 46/9% را نشان داد. تغییرات میانگین HDL-c وLDL-c  پایه، روزهای 45 و 90 اختلاف معنی‌داری داشته و به ترتیب کاهش 33/8% و 80/9% را نشان ‌داد. بنابراین مصرف چای‌ترش می‌تواند در پیشگیری از بیماری‌های قلبی‌عروقی مفید باشد. نتیجه‌گیری: در مطالعه حاضر کاهش HDL-c اختلاف معنی‌داری بین دو گروه نشان داد که از نظر بالینی معنی‌دار نبود، در سایر لیپیدها اختلاف معنی‌داری بین دو گروه مشاهده نشد.

Title: بررسی اثر مصرف رژیم با دانسیته پایین برروی حفظ وزن کاهش‌یافته و خطر بروز بیماری‌های قلبی و عروقی

http://hsr.mui.ac.ir/index.php/jhsr/article/view/776

مقدمه:اطلاعات اندکی‌ درمورد اثر رژیم با دانسیته پایین بر روی حفظ وزن کاهش یافته و خطر بروز بیماری‌های قلبی و عروقی وجود دارد. بدین منظور، مطالعه حاضر به بررسی اثر مصرف رژیم با دانسیته پایین انرژی برروی حفظ وزن کاهش یافته و خطر بروز بیماری‌های قلبی و عروقی به صورت همزمان پرداخته است. روش‌ها:۷۰ نفر که طی یک سال گذشته رژیم کاهش وزن داشته‌اند در مطالعه کار آزمایی بالینی حاضر با طراحی موازی شرکت کردند. گروه نمونه رژیم با دانسیته پایین شامل ۳۰٪ چربی‌، ۱۵٪ پروتئین و ۵۵ ٪ کربوهیدرات و گروه شاهد رژیم معمولی شامل ۳۵٪ چربی‌، ۱۵٪ پروتئین و ۵۰٪ کربوهیدرات به مدت ۷ ماه دریافت کردند. ثبت ۳ روزه‌ی غذا به منظور ارزیابی دریافت‌های غذایی استفاده شد. مارکرهای بیوشیمیایی و تن‌سنجی بر اساس پروتوکول اندازه‌گیری شدند.  یافته‌ها: وزن در گروه نمونه با دانسیته پایین انرژی۴/0±۳/۱٪ و 2/0±۳/٠-٪ (0٠٢/0=P) در مقایسه با گروه شاهد به طور معناداری کاهش پیدا کرد. نتیجه مشابهی در رابطه با دور کمر۱/0±۳/٪٠و 2/0±۴/٠-٪ (05/0>P) نیز دیده شد. قند خون ناشتا۱±۴/٠و ٨/0±٥/٩-(0٠٠۱/0=P) در گروهی که رژیم با دانسیته پایین دریافت می‌کردند نیز کاهش پیدا کرد. درصد تغییرات سطح کلسترول۴/٠±٠٥/٢و ٥/0±۴/٠-(۴0/0=P) وLDL-c در گروه دریافت‌کننده رژیم با دانسیته پایین ٩/0±۳/٠-و ٩/0±٨/۴ (0٠٢/0=P) در مقایسه با گروه شاهد به طور معناداری کاهش یافت. نتیجه‌گیری: اثرات مفید رژیم با دانسیته پایین بر روی حفظ وزن کاهش یافته در افرادی که رژیم کاهش وزن دریافت می‌کردند در مطالعه حاضر مشخص شد که ممکن است به دلیل افزایش دریافت میوه‌جات، سبزیجات و غلات کامل باشد.

Title: ارتباط مصرف کربوهیدرات رژیم غذایی و فنوتیپ دور كمر هيپرتري گليسريدميك در زنان معلم شهرستان لنجان

http://hsr.mui.ac.ir/index.php/jhsr/article/view/1433

مقدمه: با وجود دریافت بالای کربوهیدرات‌ها در رژیم غذایی ایران‌ها و شیوع بالاتر فنوتیپ دور كمر هيپرتري گليسريدميك در زنان ایرانی تاکنون مطالعه‌ای در زمینه ارتباط دریافت کربوهیدرات با این فنوتیپ در کشور انجام نشده است. این مطالعه با هدف بررسی ارتباط دریافت کربوهیدرات رژیم غذایی با فنوتیپ دور كمر هيپرتري گليسريدميك به انجام رسیده است. روش‌ها: مطالعه حاضر یک مطالعه مقطعی بود که بر روی 442 زن معلم 60-20 ساله شهرستان لنجان انجام شد. شاخص‌های تن‌سنجی، فشار خون، شاخص‌های بیوشیمیایی و فعالیت فیزیکی در تمام افراد مورد مطالعه ارزیابی گردید. با استفاده از پرسشنامه نیمه کمی بسامد خوراک dish based semi-quantitative food frequency questionnaire (DFQ)، دریافت‌های غذایی افراد در طی سال گذشته بررسی شد، سپس پنجک‌های دریافت کربوهیدرات تعیین گردید. یافته‌ها: شیوع فنوتیپ دور كمر هيپرتري گليسريدميك در زنان معلم 9/18% بود. ارتباط معني‌داری بین پنجک‌های دریافت كربوهيدرات رژيم غذايي با فنوتیپ دور كمر هيپرتري گليسريدميك دیده شد. نتیجه‌گیری: بین کربوهیدرات رژیم غذایی با فنوتیپ دور كمر هيپرتري گليسريدميك پايين كربوهيدرات ارتباط معنی‌داری دیده شد. انجام مطالعات آینده نگر در این زمینه ضرورت دارد.

Title: بررسی اثر مصرف سالاد و ماست به عنوان پیش غذا بر کنترل وزن و عوامل خطر بیماری‌های قلبی- عروقی

http://hsr.mui.ac.ir/index.php/jhsr/article/view/769

مقدمه: اگرچه مطالعات پیشین اثر کاهندگی مصرف پیش‌غذا بر انرژی دریافتی در یک وعده غذایی را نشان داده‌اند، تا کنون مطالعه‌ای اثر طولانی مدت مصرف پیش‌غذا بر وزن بدن و عوامل خطر بیماری‌های قلبی-عروقی را بررسی نکرده است. روش‌‌ها: مطالعه حاضر در قالب یک کارآزمایی بالینی موازی بر روی 60 فرد (35 زن و 25 مرد) انجام گرفت. افراد به صورت تصادفی به مدت 3 ماه از رژیم‌های غذایی کم انرژی و مشابه از نظر ترکیب درشت مغذی‌ها تبعیت کردند. افراد در گروه پیش‌غذا، 15 دقیقه قبل از وعده غذایی اصلی سالاد سبزیجات، ماست و آب مصرف می‌نمودند و در گروه کنترل، سالاد سبزیجات و ماست را همزمان با غذای اصلی مصرف می‌کردند. یافته‌ها: : با وجود تجویز مقادیر مشابهی از انرژی و درشت مغذی‌ها، انرژی و کربوهیدرات دریافتی در گروه کنترل بیشتر از گروه پیش‌غذا بود. وزن و دور کمر در گروه پیش‌غذا به ترتیب ٪5/0±8/7- و ٪2/0±7/2- و در گروه کنترل ٪59/0±6/4- و ٪1/0±6/1- کاهش یافتند (05/0>P). در گروه پیش‌غذا تری‌گلیسرید، کلسترول تام و فشار خون سیستولیک به ترتیب ٪1/1±7/5-، ٪53/0±1/3- و ٪4/0±4/4- (در تمام موارد: 05/0>P) و در گروه کنترل به ترتیب ٪9/0±2/2-، ٪59/0±2/1- و ٪3/0±6/2- کاهش یافتند. قند خون ناشتا و LDL-c فقط در گروه پیش‌غذا به طور معنی‌دار کاهش یافتند (٪1/7- در مقابل ٪0/1- و ٪03/4- در مقابل ٪6/1- ؛ 05/0>P). نتیجه‌گیری: مصرف سالاد، ماست و آب به عنوان پیش‌غذا اثرات مفیدتری بر شاخص‌های تن‌سنجی و عوامل خطر بیماری‌های قلبی-عروقی نسبت به مصرف همزمان آن‌ها با غذا در افراد اضافه وزن و چاق دارد.

Title: الگو و اولویت‌های مصرف گوشت در خانواده‌های متوسط شهری– اصفهان، ایران

http://hsr.mui.ac.ir/index.php/jhsr/article/view/1129

مقدمه: گوشت پر هزينه‌ترين بخش سبد غذايي را در هر جامعه‌ايي تشكيل مي‌دهد و مصرف آن در جوامع غني و ضعيف تفاوت قابل ملاحظه‌اي دارد. سياست كلي در سال‌هاي اخير جهت مبارزه با بیماری‌های وابسته به مصرف گوشت، گسترش مصرف گوشت سفيد به خصوص گوشت ماهی نسبت به گوشت قرمز بوده است. با اين حال مشخص نيست كه در جامعه کلان شهر سلايق خانواده‌ها و تمايل آنها تا چه اندازه تغيير كرده است. روش‌ها: این بررسی یک مطالعه مشاهده‌ای و توصیفی است که به صورت مقطعی انجام شد. تعداد نمونه مورد مطالعه 1067 برآورد شد. جامعه مورد بررسی خانواده‌هایی از شهر اصفهان بود که طی نمونه‌گیری به شکل خوشه‌ایی مرحله‌ای وارد مطالعه شدند. تجزیه و تحلیل نتایج با استفاده از نرم افزار SPSS و آمار توصیفی برای وصف فراوانی و شاخص‌های مرکزی شاخص‌های مورد پرسش در جامعه صورت گرفت و از تجزیه واریانس یک سویه و آزمون  LSDجهت مقایسه میانگین‌ها در چارک‌های مصرف استفاده شد. یافته‌ها: نرخ پاسخ در این طرح، 6/62٪ بود.از افراد مورد پرسش(8/60٪) گوشت گوسفند را به عنوان اولین اولویت برای تهیه غذا در منزل انتخاب کنند. گوشت مرغ، ماهی و گاو در رده‌های بعدی اولویت مصرف قرار گرفتند. میانگین مصرف خانگی برای سه نوع گوشت قرمز، گوشت مرغ و ماهی به ترتیب 62/21 ±( 46 /20)،30/29± (61/24) و 78/2 ±( 64 /2) برآورد گردید. از شاخصه‌های دموگرافیک تعداد افراد خانوار اثر مشخصی در میزان مصرف گوشت قرمز و مرغ داشت به طوری که در خانواده‌های 1 تا 4 نفره نسبت بیشتری از جمعیت در چارک‌های پر مصرف قرار گرفتند اما در خانواده‌های بیش از چهار نفر نسبت بیشتری از افراد در چارک‌های ابتدایی مصرف واقع می‌شوند.  نتیجه‌گیری: این مطالعه نشان داد که نسبت به مطالعات قبلی تمایل بیشتری برای مصرف ماهی و ورود آن به الگوی غذایی وجود دارد. با این حال میزان مصرف خانگی آن نسبت به میانگین مصرف جهانی فاصله زیادی دارد. علاوه بر این، سلایق سنتی برای استفاده از گوشت گوسفند همچنان پا برجاست. از شاخص های دموگرافیک ، تحصیلات و شغل افراد مسؤول تهیه غذا در خانوارهای مورد بررسی تاثیری بر الگوی مصرف گوشت قرمز و ماهی نداشت. علی‌رغم ذکر کم اهمیت بودن مسایل اقتصادی جهت تهیه غذای گوشتی در خانه در پرسشنامه، معنی‌داری اثر تعداد افراد خانوار در میزان مصرف گوشت مرغ و گوشت قرمز نشان‌دهنده اهمیت اثر مسایل اقتصادی در میزان مصرف آن است.

Title: بررسی الگوی مصرف آنتی‌بیوتیک‌ها در تولید فراورده‌های غذایی با منشا دام و طیور در ایران و مقایسه آن با سایر کشورها

http://hsr.mui.ac.ir/index.php/jhsr/article/view/613

مقدمه: آنتی‌بیوتیک‌ها مهمترین دسته داروها هستند که به دلایل درمانی، پیشگیری و یا ارتقای رشد در امور دامپزشکی مصرف می‌شوند و باقیمانده‌های آنها از طریق فراورده های دامی به مصرف‌کنندگان منتقل شده و مشکلاتی را در صنعت و سلامت انسان ایجاد می‌کنند. بر این اساس این تحقیق سعی نموده است تا برای اولین بار در ایران وضعیت مصرف آنتی‌بیوتیک‌هایی که در دامپزشکی استفاده می‌شوند را مورد بررسی قرار دهد. روش‌ها: اطلاعات مورد نیاز به وسیله پرسشنامه و مکاتبه جمع‌آوری گردید. برای تعیین الگوی مصرف داروهای آنتی‌بیوتیک پرسشنامه‌ای تهیه و با 9 نفر کلنسین‌های دامپزشکی و 13 داروخانه دامی و 3 شرکت توزیع کننده داروهای دامی که در استان چهارمحال و بختیاری فعالیت دارند، مصاحبه گردید. جهت تعیین میزان مصرف داروهای آنتی بیوتیک با سازمان دامپزشکی ایران مکاتبه شد و برای تعیین میزان تولید گوشت، شیر و تخم مرغ در سال 1389 از اطلاعات موجود در وب سایت وزارت جهاد کشاورزی استفاده گردید. داده‌های اخذ شده به وسیله نرم‌افزار SPSS نسخه 19 آنالیز شد. یافته‌ها: این مطالعه نشان داد که به ازای تولید یک کیلوگرم گوشت قرمز و شیر 4/107و برای تولید گوشت مرغ و تخم مرغ 5/249 و به طور میانگین برای تولید یک کیلوگرم گوشت، شیر و تخم مرغ 133 میلی‌گرم آنتی‌بیوتیک در مزارع دام و طیور ایران در سال 1389 مصرف گردید. مقایسه اعداد به دست آمده با اعداد مشابه از سایر کشورها نشان داد که  فاکتور مصرف آنتی‌بیوتیک در ایران بالاتر از کشورهای توسعه یافته است. نتیجه‌گیری: یافته‌های این مطالعه می‌تواند برای مسؤولین امنیت و ایمنی مواد غذایی آگاهی بخش بوده تا زمینه برقراری برنامه‌های ملی برای کنترل و پایش دقیق‌تر آنتی‌بیوتیک‌ها در مواد غذایی با منشا حیوانی در ایران فراهم گردد.

Title: بررسی محتوای نگهدارنده ناتامایسین در دوغ‌های تولیدی کارخانجات لبنی با وضعیت بهداشتی متفاوت در استان اصفهان

http://hsr.mui.ac.ir/index.php/jhsr/article/view/1092

مقدمه: ناتامایسین نگهدارنده‌ای است که جهت کنترل کپک و مخمر در دوغ استفاده می‌شود در حالی که براساس قوانین سازمان غذا وداروی ایران استفاده از هرگونه نگهدارنده در دوغ ممنوع است. بنابراین هدف از این مطالعه بررسی محتوای نگهدارنده ناتامایسین در دوغ‌های تولیدی کارخانجات لبنی با وضعیت بهداشتی متفاوت در استان اصفهان بود.. روش‌‌ها: اين مطالعه مقطعي با هدف بررسی وضعیت مصرف ناتامایسین در استان اصفهان در تابستان 1391 در19 کارخانه لبنی تولید‌کننده دوغ با وضعیت بهداشتی متفاوت با استفاده از دستگاه HPLC انجام گرفت. جهت ارزیابی وضعیت بهداشتی کارخانجات لبنی تولیدکننده دوغ از فرم ارزیابی برنامه‌های پیش نیازی مصوب وزارت بهداشت، درمان و آموزش پزشکی استفاده گردید. قسمت اول فرم؛ شامل 8 سؤال مربوط به بهداشت کارگران، قسمت دوم؛ 17 سؤال مربوط به تولید و فراوری، قسمت سوم؛ 8 سؤال مربوط به شست‌وشو/ ضدعفونی/ نظافت و قسمت چهارم؛11سؤال مربوط به شناسایی خطر و پایش است. پس از تکمیل فرم، کارخانجات به چهار گروه از نظر وضعیت بهداشتی به صورت کاملا"مطلوب، مطلوب، متوسط و نامطلوب تقسیم گردیدند. یافته‌ها: نتایج توصیفی این مطالعه نشان‌دهنده وجود ناتامایسین در چهار نمونه از دوغ‌های تولیدی در کارخانجات لبنی تولید‌کننده دوغ با وضعیت بهداشتی متوسط بود. بر اساس نتایج حاصل از فرم؛ به ترتیب، 3 کارخانه (7/15%)، 6 کارخانه (5/31%)، 6 کارخانه (5/31%)، 4 کارخانه (3/21%) از وضعیت بهداشتی کاملا" مطلوب، مطلوب، متوسط و نامطلوب برخوردار بودند.  نتیجه‌گیری: پیشنهاد می‌گردد که به کارگیری تدابیر بهداشتی در امر تولید و فراوری به همراه به کارگیری پیش نیازهای مربوط به شناسایی خطر و پایش در راستای اجرای سیستم HACCP، با دقت بیشتری در کارخانجات تولید‌کننده غذایی انجام گیرد تا بتوان کنترل آلودگی میکروبی در محصولات غذایی را بدون استفاده از نگهدارنده‌ها عملی نمود.

Title: بررسی عوامل مرتبط با مصرف میوه و سبزیجات در دانش‌آموزان دبیرستانی شهر اصفهان با استفاده از تئوری رفتار برنامه‌ریزی‌شده در سال تحصیلی 1392-1391

http://hsr.mui.ac.ir/index.php/jhsr/article/view/1186

مقدمه: امروزه در میان نوجوانان، سبک زندگی کم‌تحرک همراه با رژیم غذایی حاوی مقادیر کم میوه و سبزیجات و ميزان بالاي کربوهیدرات پیچیده و چربی به طور چشمگيري افزایش یافته است. این رفتارهای ناسالم با چاقی، دیابت نوع دو، فشار خون بالا و بیماری‌های قلبی عروقی در ارتباط می‌باشد. هدف از این مطالعه تعیین عوامل مرتبط با مصرف میوه و سبزی در میان دانش آموزان دبیرستانی با استفاده از تئوری رفتار برنامه‌ریزی شده (TPB) جهت شناخت متغیرهای موثري که در مداخلات مورد توجه قرار می‌گیرد، می‌باشد.              روش‌ها: در یک مطالعه توصیفی – تحلیلی در سال تحصیلی 1392-1391 با استفاده روش نمونه‌گیری چندمرحله‌ای، 648 دانش‌آموز دبیرستانی که پرسشنامه مبتنی بر سازه‌های تئوری رفتار برنامه‌ریزی‌شده شامل: نگرش، هنجارهای انتزاعی، کنترل رفتاری درک‌شده، قصد و مصرف میوه و سبزیجات را تکمیل نمودند، انتخاب شدند. آنالیز آماری داده‌ها با استفاده از آزمون‌های توصيفي، همبستگی اسپیرمن، من ویتنی، کروسکال والیس و رگرسیون خطی انجام شد. یافته‌ها: میانگین سنی آزمودنی‌ها 63/15 با انحراف معیار 02/1 بود. نتایج مطالعه نشان داد میانگین مصرف روزانه میوه‌ها و سبزیجات در جنس دختر به ترتیب 75/1 و36/1 و در پسران 46/1 و24/1 در روز بود. اجزای تئوری با هم دیگر به خوبی قصد مصرف میوه و سبزیجات را در دانش‌آموزان پیش‌بینی می‌کردند که ميزان R2 حدود43% به دست آمد. کنترل رفتاری درک‌شده و سپس نگرش و هنجارهای انتزاعی مهم‌ترین متغیرهای پیش‌گویی‌کننده قصد بودند. نتیجه‌گیری: یافته‌های این مطالعه نشان می‌دهد که ارتقا کنترل رفتاری درک‌شده دانش‌آموزان برای افزایش مصرف میوه و سبزیجات باید به عنوان اولویت‌های برنامه‌های ارتقا‌دهنده سلامت در میان دانش‌آموزان در نظر گرفته شود. همچنین نتایج حاکی از آن می‌باشد که تئوری رفتار برنامه‌ریزی‌شده می‌تواند به عنوان یک چهارچوب مناسب جهت افزایش رفتارهای تغذیه‌ای سالم از جمله مصرف میوه و سبزیجات برای نوجوانان به کار رود.

Title: اثر ضخامت خمیر و پیش تخمیر بر کیفیت خمیر منجمد سنگک و نان حاصل از آن

http://hsr.mui.ac.ir/index.php/jhsr/article/view/1396

مقدمه: تکنولوژي خمير منجمد از جمله تمهيداتي است که دست‌يابي به نان با کيفيت و تازه را با حداقل امکانات و تخصص امکان‌پذير می‌سازد. اين تحقيق با هدف بررسی اثر سرعت انجماد و پيش تخمير بر خصوصيات کيفي خمير منجمد سنگک و نان حاصل از آن انجام گردید. روش‌ها: مرحله پيش تخمير بين صفر تا 120 دقيقه (زمان تخمير کامل)، با فواصل زماني 30 دقيقه انجام شد و زمان تخمير نهايي پس از انجماد برابر با اختلاف زمان پيش تخمير و تخمير کامل منظور گرديد. خمير سنگک به صورت ورقه‌هایی با ضخامت‌های 3، 6 و یا 9 میلی متر شکل داده شد و پس از انجام پیش تخمیر در 25- درجه سانتی‌گراد منجمد شدند. خمير منجمد سنگک پس از 24 ساعت نگه‌داري در دماي 18- درجه يخ‌زدايي شده و پس از طي تخمير نهايي پخت گرديد. بررسي‌هاي کيفي شامل اندازه‌گيري درصد مخمر زنده مانده پس از يخ‌زدايي خمير منجمد و اندازه‌گيري دانسيته نان حاصل با آرایش فاکتوریل در قالب طرح کاملاً تصادفی صورت گرفت (05/ 0 P <). یافته‌ها: نتايج نشان داد که زنده ماني مخمر با کاهش ضخامت خمیر افزایش داشته و بیشترین زنده ماني در ضخامت سه میلی‌متر مشاهده گرديد. زمان کوتاه پيش تخمير (30 دقيقه) بالاترين شاخص زنده ماني مخمر پس از انجماد را نشان داد. کم‌ترین دانسیته نان نیز در پیش تخمیر 30 دقیقه و ضخامت سه میلی‌متر حاصل گردید. نتیجه‌گیری: به طور کلی انجماد اثر منفی بر خصوصیات کیفی خمیر منجمد و نان حاصل نشان داد. با انجام پيش تخمير کوتاه و ضخامت خمیر کمتر، می‌توان شرایط بهینه‌ای جهت تولید خمیر منجمد سنگک فراهم نمود.

Title: بررسی آلودگی به آفلاتوکسینM1 در پنیرهای سفید ایرانی شهر اصفهان به روش الایزا

http://hsr.mui.ac.ir/index.php/jhsr/article/view/1399

مقدمه: آفلاتوکسین‌ها از مهمترین گروه‌های مایکوتوکسین‌ها هستند که توسط گونه‌های قارچ آسپرژیلوس نظیر آسپرژیلوس  فلاووس و آسپرژیلوس پارازیتیکوس در مواد غذایی آلوده به این  قارچ‌ها تولید می‌شوند. آفلاتوکسین M1 دفع شده در شیر حیوان‌های شیرده حاصل تغذیه خوراک آلوده به آفلاتوکسین  B1می‌باشد. هدف از انجام این مطالعه تعیین میزان آفلاتوکسین M1 در پنیر سفید ایرانی تولید شده در کارخانجات تولید کننده فراورده‌های لبنی شهر اصفهان می‌باشد. روش‌ها: درمجموع ۵٠ نمونه پنیر به طور تصادفی از پنج کارخانه این شهر جمع آوری شد و میزان آفلاتوکسین M1 در آنها با استفاده از روش الایزا مورد ارزیابی قرار گرفت. یافته‌ها: نتایج بررسی آفلاتوکسین M1 در ۵٠ نمونه پنیر سفید آزمایش شده غلظتی بین ۷٢ تا ۲٩۷ نانوگرم در کیلوگرم را نشان داد. ٣٣ نمونه از۵٠ نمونه پنیر مورد آزمایش (۶۶٪) آلوده به آفلاتوکسین M1 بوده و میانگین میزان آفلاتوکسین در نمونه های آلوده ۷/١۶۲ نانوگرم در کیلوگرم تعیین گردید. همچنین میزان آفلاتوکسین M1 در۶ نمونه (١٨/١٨٪ موارد مثبت) بیش از حداکثر تعیین شده (٢۵٠ نانوگرم در کیلوگرم) استاندارد کشورهای اروپایی بدست آمد. تجزیه و تحلیل آماری هیچ اختلاف معنی داری (٠۵/٠P<) میان میانگین میزان آفلاتوکسین M1 در نمونه های پنیر بررسی شده را نشان نداد. نتیجه‌گیری: سلامتی انسان با مصرف شیر و فراورده های لبنی آلوده به آفلاتوکسین M1 همواره مورد توجه بوده است . بنابراین مواد غذایی بایستی همواره از لحاظ میزان آفلاتوکسین بررسی شده و تا حد امکان از آلودگی به قارچ ها دور نگه داشته شوند.

Title: بررسی قابلیت کاهش کلسترول توسط سويه لاكتوباسيلوس پلانتاروم A7 (KC355240.1)با قابليت پروبيوتيكي در محیط آزمایشگاهی

http://hsr.mui.ac.ir/index.php/jhsr/article/view/1385

زمینه و هدف: هرچند کاهش كلسترول غذا از راه های مؤثر كاهش كلسترول خون است، اما اخیرا در بخش چرب شیر اجزاء مؤثر در سلامتی، با خصوصیات زیست فعالی شناسایی شده اند كه با كاهش مصرف چربی لبنی، دسترسی به منبع مهمی از ویتامین های محلول در چربی و اجزاء زیست فعال چرب شیر محدود می گردد. مطالعات نشان داده كه رشد گروهی از باكتری های لاكتیك در شیر می تواند در كاهش بخش مضر چربی شیر مؤثر باشد. جهت معرفی سویه لاكتوباسیلوس پلانتاروم A7 (که دارای خصوصیات پروبیوتیكی مختلفی است) به عنوان سویه ای بومی با قابلیت كاهش كلسترول، اطلاع از مكانسیم عمل آن ضروری به نظر می رسید. روش کار:  سویه مورد نظر در محیط کشت MRS و شیر پرچرب حاوی 70 تا 100 میکروگرم در هر میلیمتر مکعب كلسترول محلول در آب کشت گردید. سپس با جداسازی باکتری ها از محیط کشت یا شیر بوسیله سانتریفوژ، جداسازی بخش غیر قابل صابونی شدن چربی و اندازه گیری اسپکتروفوتومتریک کلسترول انجام گردید. يافته ها: با توجه به نتایج به دست آمده مشخص شد که تفاوت در نوع کلسترول استاندارد می تواند بر روش اندازه گیری آن موثر باشد. همچنین غلظت کلسترول در طول دوره رشد باکتری در محیط تغییری نکرده و خواص این باکتری در کاهش قابل توجه کلسترول و تری گلیسیرید در سرم موش های آزمایشگاهی، با فعالیت این باکتری در محیط خارج سلولی که طی آن جذب ، هضم و یا اتصال قوی یا رسوب کلسترول بر سطح باکتری را می توان رد یابی کرد، هماهنگی ندارد. نتيجه گيري:  این پژوهش نشان داد که روش مورد استفاده در مطالعات مشابه برای اندازه گیری کلسترول در محیط کشت آزمایشگاهی روش کاملا دقیقی نیست و در صورتیکه تفاوت بین سویه های مورد آزمایش در توانایی کاهش کلسترول کمتر از 10 تا 20 میلی گرم در لیتر باشد، با این روش به سختی می توان سویه های برتر را انتخاب نمود. همچنین شاید بتوان گفت که مکانیسم های دیگری مانند تولید اسید های چرب با زنجیره کوتاه که در متابولیسم کلسترول در بدن اختلال ایجاد می کنند، عامل اصلی تغییرات اثر بخش این سویه در موش های آزمایشگاهی بوده است.

Title: خواص ضد باکتریایی اسانس مرزه بختیاری بر جمعیت میکروبی گوشت چرخ شده گاو تلقیح شده با سودوموناس آئروژینوزا طی دوره نگهداری در یخچال

http://hsr.mui.ac.ir/index.php/jhsr/article/view/1383

مقدمه: امروزه، تلاش برای کاهش مواجهه انسان با ترکیبات نگهدارنده  شیمیایی با قابلیت خطرآفرینی بالا، موجب شده است تا در تعداد زیادی از مطالعات، کاربرد ترکیبات طبیعی در نگهداری غذا و جایگزینی آنها با ترکیبات شیمیایی مورد توجه قرار گیرد. هدف از انجام این مطالعه بررسی اثر ضدمیکروبی اسانس مرزه بختیاری در افزایش مدت ماندگاری گوشت در دمای یخچال طی دوره نگهداری گوشت گاو است. روش‌ها: اثر ضد میکروبی اسانس مرزه بختیاری با سه غلظت 025/0، 05/0 و 1/0٪ بر جمعیت سودوموناس آئروژینوزا و بار میکروبی کل گوشت خام گاو تلقیح شده با میکروب طی نگهداری در دمای یخچال به مدت 14 روز ارزیابی شد. یافته‌ها: تیمار گوشت حاوی غلظت 1/0٪ اسانس مرزه بختیاری بیشترین فعالیت میکروبی را در مقابل رشد سودوموناس آئروژینوزا و بار میکروبی کل نشان داد در حالی که قدرت بازدارندگی در همه تیمارهای حاوی اسانس در کل دوره نگهداری بیشتر از تیمار کنترل بود. نتیجه‌گیری: کاربرد اسانس مرزه بختیاری با مواد نگهدارنده طبیعی به عنوان جایگزین نگهدارنده‌های سنتزی در گوشت و فراورده های گوشتی سرد به منظور افزایش عمر ماندگاری گوشت امکان پذیر است.

Title: بررسی اثر مصرف دانه خرفه بر شاخص‌های گلیسمیک و فراسنج‌های لیپیدی سرم در بیماران دیابتی نوع دو: کارآزمایی بالینی متقاطع

http://hsr.mui.ac.ir/index.php/jhsr/article/view/1148

مقدمه: اطلاعات محدودی در مورد اثر خرفه بر دیابت وجود دارد. اکثر مطالعات قبلی اثرات سودمند خرفه در مدل‌های حیوانی را نشان داده‌اند. مطالعه حاضر با هدف ارزیابی اثر دانه خرفه بر وضعیت گلایسمیک و پروفایل لیپیدی در افراد دیابتی نوع 2 انجام شد. روش‌ها: این کار آزمایی بالینی متقاطع روی 48 فرد دیابتی انجام گرفت. شرکت‌کنندگان به صورت تصادفی به یکی از دو گروه مداخله که روزانه 10 گرم دانه خرفه آسیاب شده همراه با 240 سی‌سی ماست کم چرب (5/1%) یا گروه کنترل که روزانه تنها 240 سی‌سی ماست کم چرب (5/1%) به مدت 5 هفته دریافت کردند، تخصیص داده شدند. پس از 2 هفته دوره پاکسازی، گروه‌ها به مدت 5 هفته جابجا شدند. در ابتدا و انتهای هر فاز نمونه‌های خون سیاهرگی ناشتا به منظور اندازه‌گیری سطح گلوکز ناشتای پلاسما، سطح سرمی انسولین و پروفایل لیپیدی جمع‌آوری شدند. یافته‌ها: میانگین سنی شرکت‌کنندگان مطالعه 4/51 بود. پس از مصرف خرفه کاهش معنی‌داری در وزن (57/0- در برابر 09/0 کیلوگرم، 003/0=P) و نمایه توده بدنی (23/0- در برابر 02/0کیلوگرم بر متر مربع،004/0=P) مشاهده شد. علی رغم کاهش جزئی گلوکز ناشتای پلاسما (10/2- در برابر 77/2- میلی گرم بر دسی لیتر، 9/0 =P) تغییر معنی‌داری در سطح سرمی انسولین و HOMA-IR پس از مصرف خرفه دیده نشد. با اینکه مصرف خرفه باعث کاهش معنی‌داری در سطح سرمی تری‌گلیسرید (5/25- در برابر 8/1- میلی گرم بر دسی لیتر، 04/0=P) شد اما بر HDL-c, LDL-c و کلسترول تام اثر معنی‌داری نداشت. بعد از 5 هفته مصرف خرفه، کاهش معنی‌داری در فشار خون سیستولیک (33/3- در برابر 5/0 میلی متر جیوه،01/0=P) و همچنین کاهش حاشیه‌ای در فشار خون دیاستولیک (12/3- در برابر 93/0- میلی‌متر جیوه، 09/0=P) مشاهده شد. نتیجه‌گیری: مصرف دانه خرفه به مدت 5 هفته در افراد دیابتی نوع دو باعث بهبود شاخص‌های تن‌سنجی، سطح سرمی تری‌گلیسرید و فشار خون شد. مطالعات بیشتری برای اندازه‌گیری مقدار مناسب مصرفی در بیماران دیابتی مورد نیاز است.

Title: بررسی اثر ضد دیابت عصاره ریشه گیاه کهور (Bank & Soland) J.F.Macbar Prosopis farcta در موش صحرایی

http://hsr.mui.ac.ir/index.php/jhsr/article/view/1372

مقدمه :دیابت یک بیماری مزمن متابولیک است که علی رغم درمان های دارویی و اختصاصی هنوز یک مشکل اساسی پزشکی محسوب می شود. گیاهان متعددی تا کنون در درمان دیابت معرفی شده اند. یکی از این گیاهان گیاه کهور (Prosopis farcta) است که به عنوان پائین آورنده قند خون در طب سنتی مصرف می شود. هدف از این مطالعه بررسی اثر پائین آورنده قند خون ریشه این گیاه در موشهای صحرائی سالم و دیابتی می باشد. روش بررسی: ریشه گیاه از مناطق جنوبی ایران جمع آوری شد. به روش پرکولاسیون عصاره گیری انجام شد. موش های صحرایی سالم به دو گروه کنترل و دریافت کننده g/kg 12 عصاره تقسیم شدند. موش های دیابتی شده با استرپتوزوتوسین به شش گروه تقسیم شدند. سه گروه عصاره با دوز های g/dl 12، 8 و 4، یک گروه گلی بنکلامید با دوز mg/kg 10، یک گروه انسولین NPH با دوز IU/kg5 و یک گروه نرمال سالین ml/kg 1(کنترل) دریافت کردند. خونگیری از دم حیوان در ساعات شروع مداخله، 1، 2، 3، 5، 7، 24 و روز هشتم صورت گرفت. قند خون توسط گلوکومتر اندازه گیری شد.   یافته ها: آزمایشات فیتوشیمیایی مقدماتی نشان داد عصاره ریشه حاوی تانن، فلاونوئید، ساپونین و آلکالوئید می باشد. عصاره ریشه گیاه با دوزهای g/dl 12، 8 و 4 در حیوانات دیابتی و سالم روی کاهش قند خون مؤثر نبود. گلی بنکلامید و انسولین قند خون را در حیوانات دیابتی به طور معنی داری کاهش داد.  نتیجه گیری: به نظر نمی رسد که عصاره ریشه گیاه بر روی کاهش قند خون مفید باشد. تحقیقات بیشتری برای شناسایی آثار دیگر و ترکیبات شیمیایی گیاه لازم است.

Title: ارتباط مصرف ماهی با سندرم متابوليک در زنان 60-20 ساله

http://hsr.mui.ac.ir/index.php/jhsr/article/view/1035

مقدمه: اگر چه مصرف ماهی ارتباط معکوسی با اختلالات متابولیک دارد ولی اطلاعات اندک و ضد و نقیضی در مورد ارتباط بین مصرف ماهی و سندرم متابولیک در دسترس می‌باشد. مطالعه حاضر با هدف بررسي ارتباط مصرف ماهی و سندرم متابوليک و اجزای آن انجام شد. روش‌ها: اين مطالعه مقطعي بر روي 420 نفر از پرستاران زن 60-20 ساله شاغل در بيمارستان‌هاي اصفهان که به روش نمونه‌گيري خوشه‌اي چند مرحله‌اي به صورت تصادفي انتخاب شده بودند، انجام شد. دريافت‌هاي غذايي معمول فرد در طي سال گذشته با استفاده از يك پرسشنامه نيمه كمي بسامد خوراك ارزيابي شد. مصرف ماهی با جمع کردن مقدار مصرف انواع ماهی از جمله ماهی تن، محاسبه شد. شاخص‌هاي تن سنجي و فشار خون طبق دستورالعمل‌هاي استاندارد ارزيابي شدند. تمام شاخص‌هاي بيوشيميايي بر روي نمونه خون ناشتا اندازه‌گيري شدند. سندرم متابوليک بر طبق معيارها III) Adult Treatment Panel) ATP III تعریف شد. يافته‌ها: میانگین دریافت روزانه ماهی 4/14 گرم در روز بود. مصرف ماهی به طور معکوسی با شیوع سندرم متابولیک ارتباط داشت.  افرادی که در بالاترین سهک مصرف ماهی قرار داشتند، از 65% شانس کمتری برای ابتلا به سندرم متابولیک برخوردار بودند. تعدیل برای عوامل مخدوش‌گر و متغیرهای غذایی باعث تقویت این ارتباط گردید. حتی وقتی BMI مورد محاسبه قرار گرفت، افرادی که در بالاترین سهک مصرف ماهی قرار داشتند، از 96% شانس کمتر برای ابتلا به سندرم متابولیک در مقایسه با افراد پایین‌ترین سهک برخوردار بودند. پس از تعدیل برای عوامل مخدوشگر افرادی که در بالاترین سهک مصرف ماهی قرار داشتند، نسبت به افراد پایین‌ترین سهک، از 89% شانس کمتری برای ابتلا به هیپرتری گلیسیریدمی، 43% شانس کمتر برای ابتلا به HDL پایین و از 77% شانس کمتر برای ابتلا به فشارخون بالا برخوردار بودند. نتيجه‌گيري: يافته‌هاي اين مطالعه حاکي از ارتباط معکوس معني‌داری بين مصرف ماهی و سندرم متابوليک و برخي از اجزاي آن بود. انجام مطالعات آينده‌نگر براي تأييد اين يافته‌ها ضرورت دارد.

Title: بررسی غلظت فلوراید دریافتی از طریق آب و چای مصرفی در ساکنین شهر قم در سال 1391؛ یک مطالعه مقطعی

http://hsr.mui.ac.ir/index.php/jhsr/article/view/1364

مقدمه: بدن انسان فلوئور را از منابع مختلفی از جمله آب و غذا دریافت می‌کند. وجود این عنصر به منظور پیشگیری از پوسیدگی دندان ضروری است. از سویی با رسیدن غلظت آن به بیش از حد مجاز منجر به آسیب‌های زیادی از جمله فلوئوروزیس دندانی و اسکلتی می‌گردد. در بین نوشیدنی‌ها چای از جمله منابع با غلظت بالای فلوراید محسوب می‌شود. بررسی حاضر با هدف سنجش غلظت فلوراید در آب و چای مصرفی شهر قم صورت گرفت. روش‌ها: پژوهش حاضر مطالعه توصیفی تحلیلی است که به صورت مقطعی در سال 1391 بر نمونه‌های آب و نشان‌های تجاری چای‌های پر مصرف در شهر قم انجام شد. غلظت فلوراید در نمونه‌های آب با استفاده از دستگاه اسپكتروفتومتر HACH مدل DR-4000 و فلوراید موجود در نمونه‌های چای با استفاده از روش یون انتخابی اندازه‌گیری شد. اطلاعات پس از جمع‌آوري با استفاده از نرم افزار SPSS نسخه 17 تجزيه و تحليل شد. یافته‌ها: با توجه به آزمایشات صورت گرفته میانگین غلظت فلوراید موجود در آب شرب بین mg/l 63/0 -07/0 به دست آمد. کمترین مقدار مربوط به دستگاه‌های تصفیه خانگی بود. غلظت فلورايد در چا ي‌هاي خشک كيسه‌اي mg/kg 9/138و در چای‌های خشک سیاه دانه mg/kg71/153 بود. همچنین غلظت این عنصر در چای‌های دم کرده کیسه‌ای mg/l 29/1 و در چای‌های دم کرده سیاه دانهmg/l 5/1 بدست آمد. نتیجه‌گیری: نتابج این بررسی نشان داد که دستگاه‌های تصفیه آب خانگی و صنعتی میزان فلوراید موجود در آب آشامیدنی را تا حد زیادی کاهش می‌دهند. بنابراین نوشیدن چای می‌تواند کمبود فلوراید موجود در آب آشامیدنی را تا حدی جبران کند.

Title: بررسی رابطه الگوهای غذایی غالب با دیابت نوع 2

http://hsr.mui.ac.ir/index.php/jhsr/article/view/990

مقدمه: دیابت به عنوان عامل خطر بیماری‌های مزمنی مانند قلبی عروقی با مرگ و میر بالا، مطرح است. شیوع دیابت در کشورهای در حال توسعه از جمله ایران رو به افزایش است.در این مطالعه رابطه الگوهای غذایی غالب بادیابت نوع 2 بررسی شد. روش‌ها: در این مطالعه مورد شاهدی 332 فرد (110 مورد با دیابت تازه تشخیص داده شده و 222 شاهد) به روش نمونه گیری آسان انتخاب شدند. دریافت های غذایی افراد با استفاده از یک پرسشنامه بسامد خوراک نیمه کمی معتبر ارزیابی گردید. شاخص های تن سنجی و فشار خون طبق دستورالعمل های استاندارد، اندازه گیری و نمایه توده بدن و نسبت دور کمر به دور باسن محاسبه گردید. نمونه خون سیاهرگی، جهت اندازه‌گیری متغیرهای متابولیکی جمع‌آوری گردید. جهت شناسایی الگوهای غذایی از روش تحلیل عاملی استفاده شد. رابطه الگوهای غذایی غالب با دیابت با روش رگرسیون لجستیک بررسی شد. یافته‌ها: سه الگوی غذایی غالب در افراد مورد مطالعه شناسایی شد: الگوی غذایی سالم (غنی از سبزی‌ها، روغن‌های گیاهی، ماهی، میوه‌ها و مغزها)، الگوی غذایی مختلط (مصرف بالای نمک، گوشت امعا و احشا، میوه‌های خشک، مرغ، چای، لبنیات کم‌چرب و برخی سبزی) و الگوی غذایی سنتی (مصرف بالای سیر، دوغ، لبنیات پر چرب، میوه های خشک، گوشت قرمز، غلات و چربی حیوانی). پس از تعدیل اثر متغیرهای مخدوشگر، افرادی که در بالاترین چارک الگوی غذایی مختلط و سنتی قرار داشتند به ترتیب از 117% (نسبت شانس 17/2 و فاصله اطمینان 95%: 0/1 تا 50/4) و 113% (نسبت شانس 13/2 و فاصله اطمینان 95%: 03/1 تا 41/4) شانس بیشتری برای ابتلا به دیابت نسبت به افراد پایین‌ترین چارک برخوردار بودند، در حالی که چه در مدل خام و چه پس از تعدیل برای متغیرهای مخدوشگر ارتباط معنی‌داری بین تبعیت از الگوی غذایی سالم با دیابت مشاهده نشد. نتیجه‌گیری: الگوهای غذایی مختلط و سنتی شانس ابتلا به دیابت را در افراد مورد مطالعه افزایش داد
